# Unseen abilities – how refugee women with disabilities experience social participation

**DOI:** 10.1093/eurpub/ckac129.605

**Published:** 2022-10-25

**Authors:** S Scheer, M Mondaca

**Affiliations:** 1 Health and Social Science, IB University of Applied Health and Social Science, Berlin, Germany; 2 Department of Neurobiology, Care Sciences and Society, Karolinska Institutet, Stockholm, Sweden

## Abstract

**Background:**

In the European context, refugee women especially those with physical disabilities are confronted by many unequal conditions that have an impact on their health and social wellbeing. To address these, it is important to understand refugees’ social participation and integration process. Furthermore, the purpose of the study is to identify refugee women's resources and their strengths in their everyday life that are related to current problems and needs.

**Methods:**

This research project is embedded in occupational science theories which include the effect of social and environmental issues on an individual's health and wellbeing. An ethnographic, longitudinal study using visual and narrative methods was used to explore the phenomena of the everyday life and social participation possibilities of five refugee women with physical disabilities living in Stockholm, Sweden. Moreover, to illustrate the process of meaning-making, the frame of intersectionality was used within the whole research process.

**Results:**

Through the lens of intersectionality, it was possible to determine gender, ableism, religion, and ethnicity issues as influencing factors concerning health and social participation. The women's narratives illustrate accessibility, e.g. public transport as a big resource in comparison to the country they came from. Still, to be a woman, a refugee, and having a disability at the same time is a challenge for social participation. One reason is less consideration of disabilities in the integration process. Having faith was mentioned as a safe place and using the public transport to independency. However, the women stress, that they want their abilities to be used instead of experiencing being limited by their disabilities.

**Conclusions:**

In conclusion, the women emphasize being engaged in the hosting community. Health and social policymakers should consider the refugee women's voices to develop integration strategies with and not for refugee women with disabilities.

**Key messages:**

• Through the lens of intersectionality, refugee women's narratives bring new insights about social participation.

• Refugee women with a disability want to engage in the hosting society and become active members.

